# Supplementary material for: Characterization of the Free and Membrane-Associated Fractions of the Thylakoid Lumen Proteome in Arabidopsis thaliana
Source: Int J Mol Sci. 2021 Jul 29;22(15):8126. doi: 10.3390/ijms22158126 (PMC8346976; doi:10.3390/ijms22158126)
Supplement: Supplementary file 1 [file ijms-22-08126-s001.zip › Supplementary Figure S1.pdf]

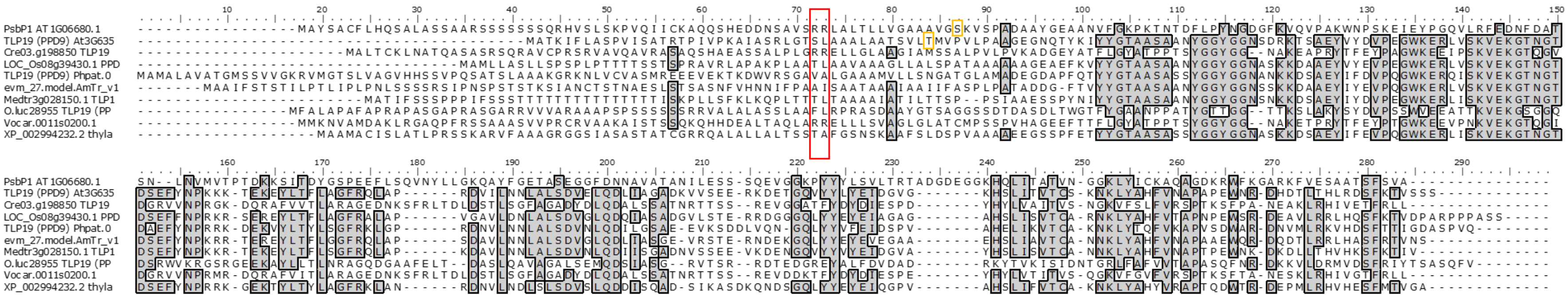

Supplementary Figure S1

Alignment of PPD9 orthologues with PsbP1 (AT1G06680) showing TAT motif (red boxes), and N-termini of PsbP and PPD9 detected in the current study (yellow boxes). Grey shading and outline indicates similar residues in the alignment
