# Supplementary material for: Characterization of the Free and Membrane-Associated Fractions of the Thylakoid Lumen Proteome in Arabidopsis thaliana
Source: Int J Mol Sci. 2021 Jul 29;22(15):8126. doi: 10.3390/ijms22158126 (PMC8346976; doi:10.3390/ijms22158126)
Supplement: Supplementary file 1 [file ijms-22-08126-s001.zip › Supplementary Figure S2.pdf]

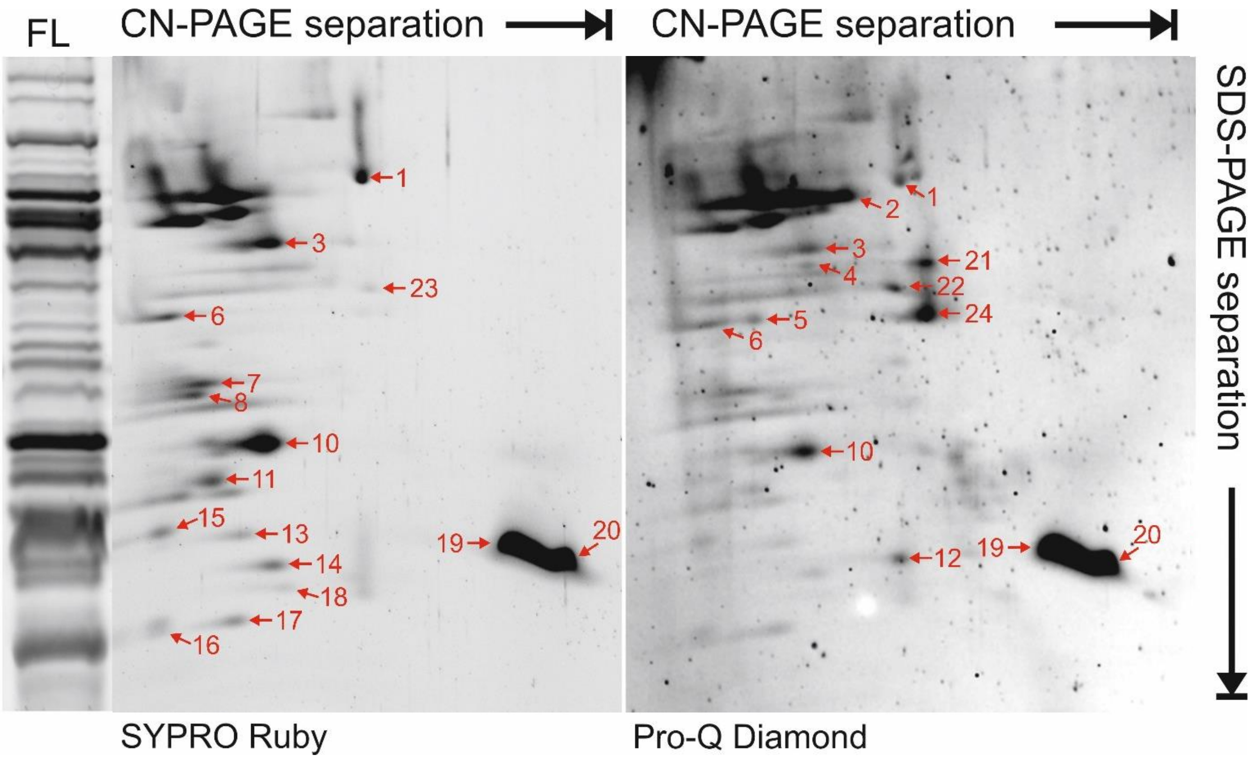

Supplementary Figure S2

Proteins from the free lumen (FL) fraction separated in two dimensions by CN-PAGE followed by SDS-PAGE. Gel was stained with SYPRO-Ruby (left) and ProQ Diamond (right), followed by silver (not shown). Numbered spots were excised and proteins identified using MS-based proteomics. Numbering of spots corresponds with numbering used in Supplemental data Table S3.
